# Development and Ex-Vivo Skin Permeation of Sildenafil Citrate Microemulsion System for Transdermal Delivery

**DOI:** 10.5812/ijpr-139381

**Published:** 2024-03-28

**Authors:** Nasibeh Jamali, Eskandar Moghimipour, Fatemeh Nikpour, Anayatollah Salimi

**Affiliations:** 1 Department of Pharmaceutics, Faculty of Pharmacy, Ahvaz Jundishapur University of Medical Sciences, Ahvaz, Iran; 2 Nanotechnology Research Center, Ahvaz Jundishapur University of Medical Sciences, Ahvaz, Iran

**Keywords:** Sildenafil Citrate, Transdermal Delivery, Microemulsion, Permeability

## Abstract

**Background:**

This study aimed to develop a microemulsion (ME)-based skin delivery platform containing sildenafil citrate (SC)-ME and evaluate its in vitro skin permeability.

**Methods:**

Accurate MEs were prepared using pseudo-ternary phase diagrams and a full factorial design with three variables at two levels. After the design phase, suitable ratios of oil, water, and a mixture of surfactant (S) and cosurfactant (CS) were selected to prepare various SC-ME formulations. These SC-MEs were analyzed for stability, droplet size, in vitro SC release, skin permeability, and viscosity properties.

**Results:**

The droplet size of the ME samples ranged from 6.24 to 32.65 nm, with viscosities between 114 to 239 cps. Release profiles indicated that 26 to 60% of SC was released from the different SC-MEs within 24 hours. All ME formulations significantly enhanced the permeability coefficient (P) through rat skin. Specifically, the flux (J_ss_) in SC-ME7 increased by approximately 117 times (J_ss_ = 0.0235 mg/cm^2^.h) compared to the control sample (0.0002 mg/cm^2^.h).

**Conclusions:**

The study concluded that the proportions of the water or oil phase and the S/CS mixture in the MEs significantly influenced the physicochemical characteristics and permeation parameters. The selected MEs improved both the permeability coefficient and the rate of permeation through rat skin. The enhanced drug delivery through and into deep skin layers is a key attribute of an ideal dermal ME. These findings suggest that MEs could serve as effective transdermal delivery systems for SC and similar drugs. However, in vivo assays and clinical research are needed to confirm the therapeutic efficacy of MEs.

## 1. Background

Due to its effectiveness and safety, sildenafil citrate (SC) is often considered the first and most effective treatment option for erectile dysfunction (ED) (1). Erectile dysfunction, a prevalent sexual disorder among men, is defined as the consistent inability to achieve or maintain an erection sufficient for satisfactory sexual intercourse (2). Nitric oxide, released by endothelial cells in response to sexual stimulation and processed in the central nervous system, activates the guanylate cyclase enzyme and the production of cyclic guanosine monophosphate (cGMP). cGMP, by lowering intracellular calcium levels, induces relaxation of the trabecular and arterial smooth muscle, leading to arterial dilation, venous constriction, and ultimately penile erection (3). Phosphodiesterase 5 (PDE5), the major isoenzyme in the cavernosum that degrades cGMP, can be inhibited by drugs like SC (4). These inhibitors prevent cGMP breakdown and facilitate smooth muscle relaxation in the corpus cavernosum, enhancing blood flow and sustaining erection (5, 6). The oral administration of SC is challenged by presystemic metabolism in the liver and significant intestinal metabolism, contributing to the side effects associated with oral SC administration (7). The cytochrome P3A4 enzyme in the liver subjects SC to oxidative biotransformation, reducing its bioavailability to 41%. Additionally, SC has been associated with several side effects, including nasal congestion, hypotension, headache, flushing, and visual disturbances (8). The efficacy of SC is notably diminished when taken orally with food, especially high-fat meals (9).

Oral administration of SC has been observed to exhibit a slow onset of action, ranging from 0.5 to 2 hours post-administration, with a half-life of approximately 4 hours. Consequently, to maintain a steady-state plasma concentration, repeated doses are required (8). Microemulsions (MEs) are thermodynamically stable mixtures of oil and water with low viscosity, stabilized by a surfactant (S) that usually combines with a cosurfactant (CS) (10, 11). Due to their low cost, minimal toxicity, enhanced stability, and ease of preparation, MEs have been proposed as an appealing alternative for topical and transdermal drug delivery, offering improved drug solubility and stability (12, 13). As colloidal systems, MEs have a high potential for drug absorption owing to their ability to enhance drug solubility. They facilitate drug penetration into the skin by increasing the thermodynamic affinity of the drug for the skin. The combination of water, oil, and S-CS mixtures, as the main components of MEs, can significantly improve drug penetration into the skin. The lipid matrix of the skin's outer layer, the stratum corneum, plays a vital role in regulating the permeability of various substances through the skin (14, 15). The stratum corneum and the lipid bilayer of the cell membrane can interact with the oil phase and S components of MEs to enhance drug delivery through the skin and increase penetration (15). Microemulsion-based formulations have been extensively studied to improve cutaneous and transdermal drug delivery (16). Given the considerably reduced efficacy of SC when administered orally, this study aimed to develop an ME-based transdermal delivery system for SC and assess its in vitro skin permeability.

## 2. Objectives

This study investigated an SC formulation based on the ME platform for transdermal drug delivery.

## 3. Methods

### 3.1. Materials

Sildenafil citrate powder was procured from Iran Daroo Co (Iran). Tween 80, Span 20, and polyethylene glycol (PG) were obtained from Merck Co. (Germany), while Transcutol P (TP) was received as a gift from Gattefosse (France). All these chemicals and solvents are of analytical grade. Additionally, fresh double-distilled water was utilized in all experiments. The dialysis bag was sourced from Kimia Teb Co (Mashhad, Iran).

### 3.2. Animals

Male adult Wistar rats weighing between 150 - 200 g were selected as animal models for this study. The research received approval from the Animal Ethical Committee of Ahvaz Jundishapur University of Medical Sciences (permit no. IR.AJUMS.ABHC.REC.1397.041).

### 3.3. Sildenafil Citrate Assay

A UV-visible spectrophotometer (Bio Wave II UV, Biochrom) was employed to determine the amount of SC incorporated into the MEs. For this analysis, SC powder was dissolved in a phosphate buffer (pH = 7.4, 0.1 M) and methanol solution (1:2 ratio), and the absorbance of the SC solution was measured at a wavelength of 289 nm, utilizing a molar extinction coefficient of 13,800. To establish the calibration curve for SC, various dilutions of SC were prepared in a water/methanol mixture (1:2 ratio), followed by measuring the sample's absorbance at a wavelength of 289 nm. The obtained calibration curve was subsequently used for SC quantification in future experiments.

### 3.4. Determination of SC Solubility

The solubility of SC in different ME components, including oleic acid (OA), TP, and the S and CS mixture of Span 20, Tween 80, and PG, was assessed. An excess amount of SC was dissolved in 5 mL of the aforementioned components with stirring at 25 ± 0.5°C and 200 rpm for 48 hours to achieve equilibrium. The mixture was then centrifuged at 10 000 rpm for 30 minutes to separate any undissolved components. The SC dissolved in each phase was extracted with methanol, and the absorbance of the SC solution in methanol was measured using a spectrophotometer at a wavelength of 289 nm (17).

### 3.5. Pseudo-Ternary Phase Diagram Construction

To develop high-precision MEs, an aqueous titration method was utilized to construct pseudo-ternary phase diagrams at room temperature. For this, two-phase diagrams were created, each with three variables at two levels. The S/CS ratio, oil phase fraction (Oil%), and water phase fraction (W%) were identified as the main variables. Based on the phase diagrams' results, eight ME formulations were prepared with mass ratios of 1:1, 3:1, and 10:1 for S and CS. Tween 80 and Span 20 were chosen as the S, PG as the CS, and a combination of OA and TP as the oil phase. To create the phase diagram, the determined ratios of S, CS and oil components were first mixed in the different mass ratios 9, 2:8, 3:7, 4:6, 5:5, 6:4, 7:3, 8:2 and 9:1. The above mixture was titrated dropwise with double distilled water at 25°C ± 2°C with moderate stirring. The formation of the MEs phase was confirmed by visual observations and the optimal MEs formulation was selected for preparation and further testing (17, 18).

### 3.6. Preparation of SC ME

The ME formulations were prepared using selected high and low levels of oil phase ratio (25%, 5%), water phase (10%, 5%), and S/CS mixture ratios of 1:3 and 1:1. To incorporate the drug into the MEs, SC was dissolved in the oil phase at a concentration of 1% and combined with the S-CS mixture. Double-distilled water was then added dropwise to the drug-containing solution and stirred at room temperature until a transparent mixture was obtained (19, 20). The compositions of the selected MEs are detailed in Table 1. 

**Table 1.** Selected Cosurfactant-Microemulsion Composition ^a^

ME Formulation	Factorial	S/CS	Oil ^b^	Water ^b^	S + CS ^b^	Drug Content ^b^
**SC-ME1**	+++	3:1	25	10	64	1
**SC-ME2**	++-	3:1	25	5	69	1
**SC-ME3**	+-+	3:1	5	10	84	1
**SC-ME4**	+- -	3:1	5	5	89	1
**SC-ME5**	---	1:1	5	10	84	1
**SC-ME6**	- - +	1:1	5	5	89	1
**SC-ME7**	- + -	1:1	25	5	69	1
**SC-ME8**	++ -	1:1	25	10	64	1

^a^ SC-ME, cosurfactant-microemulsion; + and – signs described high and low levels of the variable amount.

^b^ Values are expressed as %.

### 3.7. ME Droplet Size Determination

The droplet size of the MEs was measured using a Nanosizer device from QUIDIX Co. (SCATTER SCOPE 1, South Korea).

### 3.8. Viscosity Determination

The viscosity of the MEs was analyzed at 25°C ± 0.5°C using a Brookfield viscometer (DV-II+Pro, USA) with spindle number 34 at a shear rate of 100 rpm (21).

### 3.9. Evaluation of the Physical Stability of MEs

The physical stability of the MEs, including thermal stability and centrifuge stress tests, was evaluated by storing the MEs at various temperatures of 4°C, 25°C, and 37°C (75% ± 5% relative humidity) as per the International Conference on Harmonisation (ICH) guidelines over a period of 6 months. The physicochemical characteristics of the MEs, such as clarity, phase separation, and particle size, were monitored after the specified time. Additionally, the MEs underwent centrifugation using a high-speed brushless centrifuge (MPV-350R, Poland) at 10,000 rpm for 30 minutes at room temperature. The physical stability of the MEs post-centrifugation was visually assessed by examining the degree of phase separation (22).

### 3.10. Ex Vivo Permeability Studies

Ex vivo skin permeability studies were conducted using rat skin with an effective contact area of 4.906 cm^2^ in Franz diffusion cells. Before the tests, skin samples were hydrated at 25°C for a full day. The skin's stratum corneum layer faced the donor section, and the hydrated skin samples were securely positioned between the donor and receptor phases of each cell. The release test was then carried out using the prepared diffusion cells (23).

### 3.11. Evaluation of In Vitro SC Release from MEs

The release of SC from the MEs was assessed using a drug release assay with Franz diffusion cells. A cellulose membrane bag containing a phosphate buffer/methanol solution (1:2) at pH 7.4 and a temperature of 37°C served as the receptor medium. Then, 4 g of the SC-ME sample was placed into the membrane bag. The diffusion cell, filled with 38 mL of receptor medium, was situated in a water bath on a magnetic hot plate and stirred at 200 rpm. Samples of 2 mL were drawn from the receptor phase at specified time intervals of 0.5, 1, 2, 3, 4, 5, 6, 7, 8, and 24 hours and analyzed using a spectrophotometry assay at 289 nm. To maintain sink conditions, an equivalent volume of the phosphate buffer/methanol solution was replenished in the receptor fluid after each sample withdrawal. The free drug MEs and a 1% SC water solution served as negative and positive controls, respectively. The quantity of drug released was calculated using the absorption data and a calibration curve. Additionally, the skin permeability release mechanism of SC-MEs was examined through various kinetic models to identify the most fitting model (24). Microemulsions without SC and a solution of SC (1%) in phosphate buffer/methanol were used as the negative and positive control samples, respectively.

### 3.12. Calculation of Permeation Data

The permeation parameters of SC-MEs were calculated by plotting the cumulative amount of SC permeated through rat skin per unit area against time. Parameters such as flux (J_ss_) (mg/cm^2^.h), permeability coefficient (P) (cm/h), apparent diffusion coefficient (D_app_) (cm^2^/h), and enhancement ratio (ER) were determined using this graph and the applicable Equations 1-4 (25).


Equation 1. $$J_{ss}=P.C$$

Equation 2. $$T_{lag}=\frac{h^{2}}{6D}$$

Equation 3. $$D_{app}=\frac{h^{2}}{{6T}_{lag}}$$

Equation 4. $$ER=\frac{J_{ss\;},P,\;D\;(with\;enhancer)}{J_{ss}\;,P,\;D\;(without\;enhancer)}$$

Where h and D represent the diffusion coefficient of the membrane thickness, C, lag time (T_lag_) and h represent the total amount of SC permeated in the donor medium to reach a steady state, and the path length of the diffusion cell, respectively.

### 3.13. Statistics Analysis

All tests were conducted in triplicate (N = 3), and results were expressed as mean ± standard deviation (SD). A one-way analysis of variance (ANOVA) was used to compare the test groups with the control, and a P-value of < 0.05 was deemed significant.

## 4. Results

### 4.1. Solubility of SC

The solubility of SC was evaluated in various components of MEs. Table 2 presents the solubility values (mg/mL) of SC in OA, TP, a mixture of OA and TP, Span 20, Tween 80, and PG, with solubility ranging from 3.88 ± 0.15 to 18.3 ± 0.33 mg/mL. The highest solubility was observed in the OA + TP mixture, as indicated in Table 2. 

**Table 2.** Solubility Values of SC in Different Components (N = 3)

Phase Type	Component	Solubility ^a^ (mg/mL)
**Oil**	Oleic acid	7.63 ± 0.48
**Oil**	Transcutol P	3.88 ± 0.15
**Oil**	Oleic acid + Transcutol P	18.3 ± 0.33
**Surfactant**	Span 20	7.22 ± 0.34
**Surfactant**	Tween 80	12.14 ± 0.45
**Cosurfactant**	PG	4.1 ± 0.58
**Surfactant mixture**	Span 20 + Tween 80 (1:1)	11.01 ± 0.15

^a^ Values are expressed as mean ± SD.

### 4.2. Phase Studies

**Figure 1.**
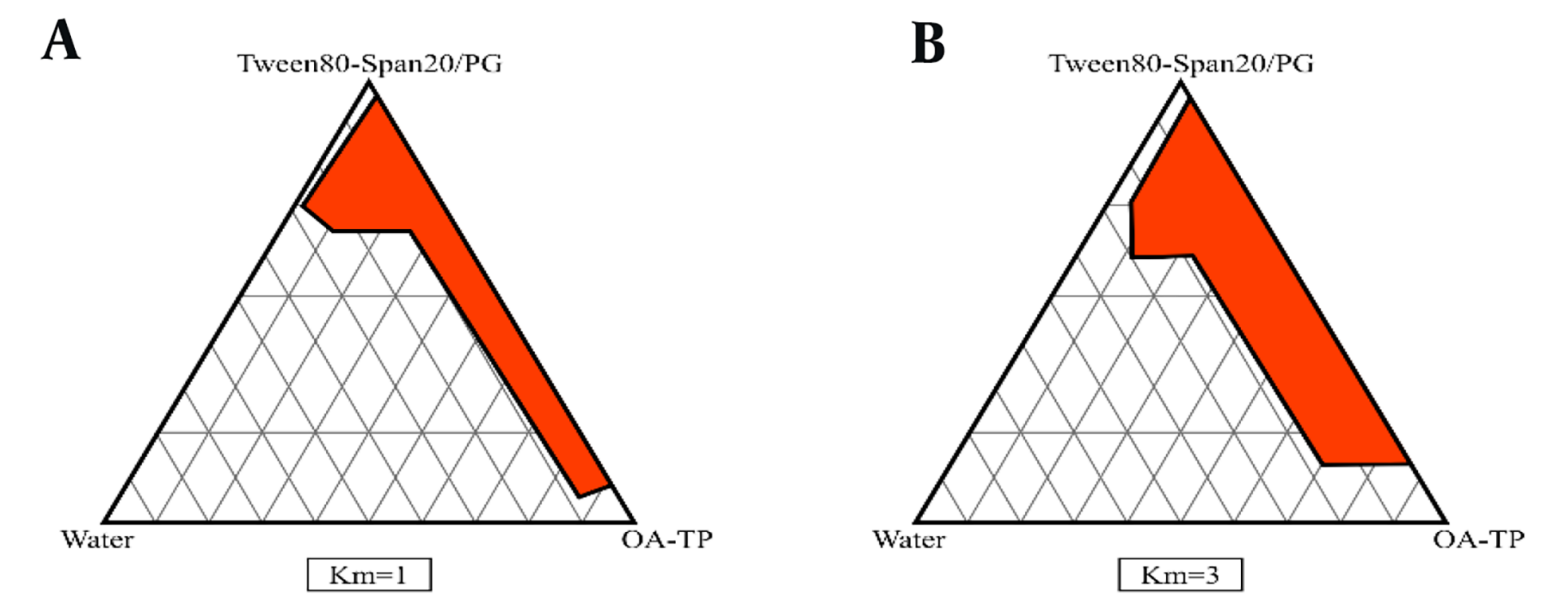
The pseudo ternary phase diagrams of oil/surfactant (S)-cosurfactant (CS) mixture/water system at the mass ratios of 1:1 and 3:1 for Tween 80 - Span 20/polyethylene glycol (PG), at ambient temperature; red area shows microemulsion (ME) zone.

The pseudo-ternary phase diagrams of different ME formulations were assessed using cross-polarized light microscopy, as shown in Figure 1. The components of the MEs, specifically oil (OA - TP), S (Span 20 - Tween 80), and cosurfactant (PG), were also titrated dropwise with water.

### 4.3. Characterization of SC-MEs

**Figure 2.**
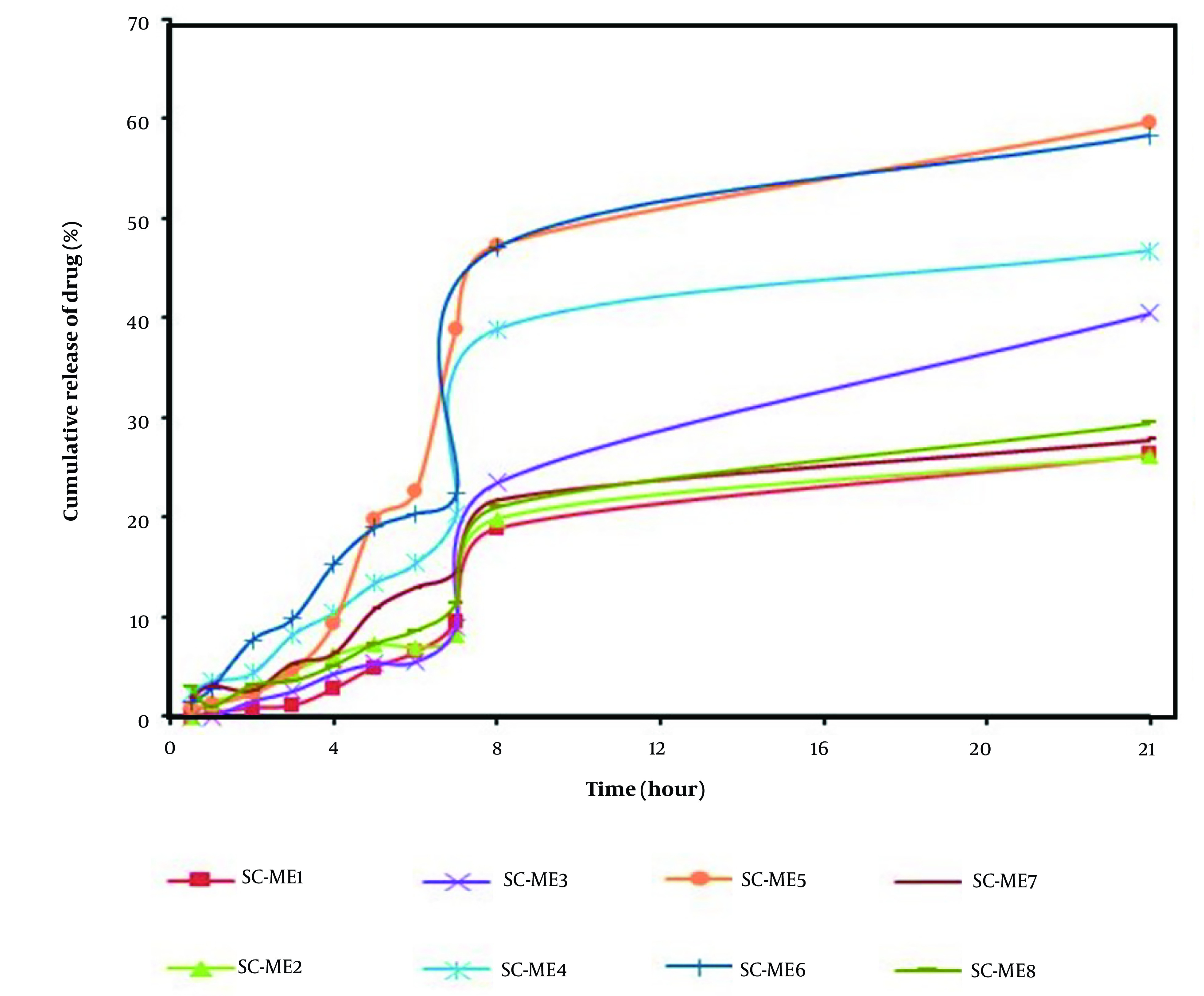
Plot of cumulative release of cosurfactant (SC) from microemulsion (ME) formulation at 24 hours

**Figure 3.**
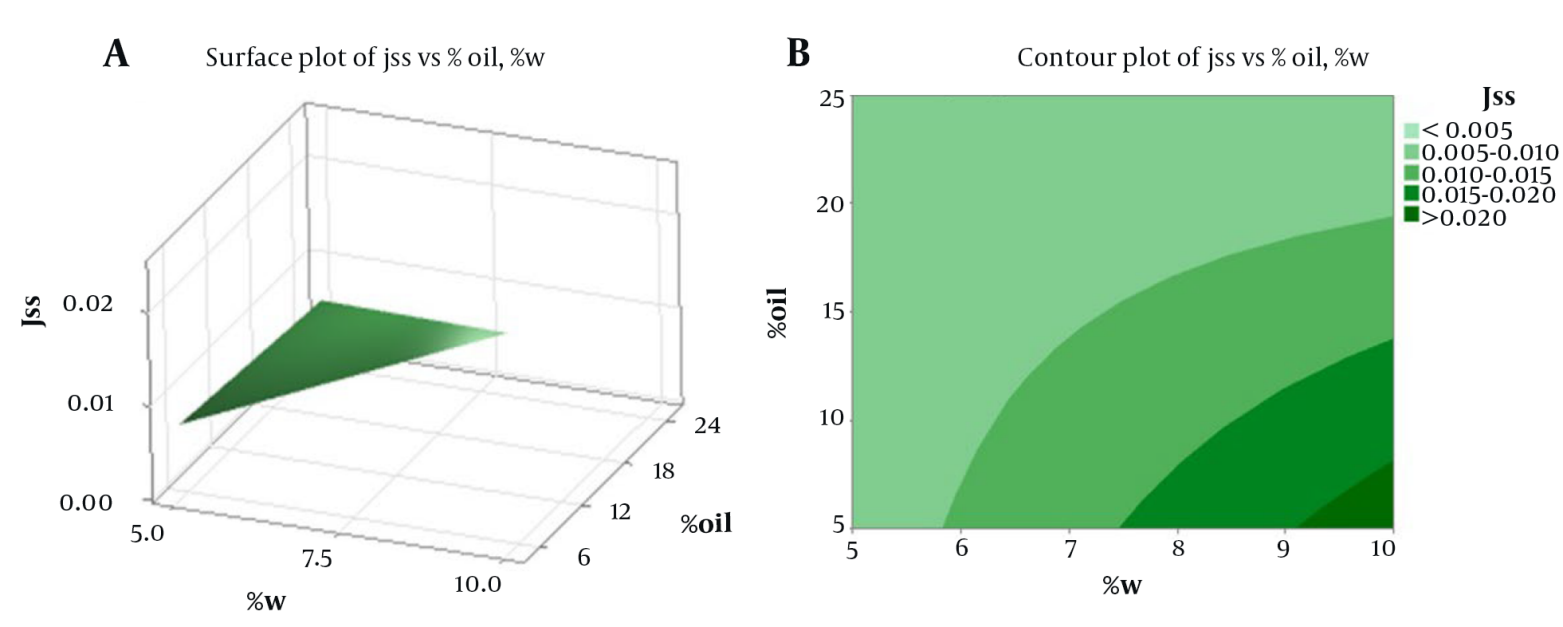
A, 3D response surface plot; and B, 2D contour plot illustrating the influence of the water and oil content on J_ss_.

Table 3 displays the physical properties of SC-MEs, including viscosity, mean droplet size, polydispersity index (PI), and pH. Additionally, Table 4 details the active ingredient content, pH value, and mean droplet size (nm) of SC-MEs after 6 months, indicating acceptable homogeneity and stability across all SC-ME formulations. Statistical analysis revealed no significant difference (P > 0.05) in droplet sizes between the beginning of the test and after six months of storage. More comprehensive data on the ME formulations, including the amount of drug released and the kinetics models of SC release, are summarized in Table 5 and Figure 2. Among the various MEs, SC-ME5, which contains 40% oil, 10% water, and 50% S + CS, showed the highest drug release of 59.58% within 24 hours (R_24 hours_). The lowest amounts of drug release, about 26% at 24 hours, were observed in SC-ME1 and SC-ME2. Furthermore, the permeability parameters of the SC-ME samples, along with 2-dimensional (2D) contour and 3-dimensional (3D) surface plots, are presented in Table 6 and Figure 3, respectively.

**Table 3.** Cosurfactant-Microemulsion Viscosity, Droplet Size, Polydispersity Index, and pH (N = 3) ^a^

MEs Code	Viscosity (cps)	Mean Droplet Size (nm)	Polydispersity Index	pH
**SC-ME1**	150 ± 0.11	6.24 ± 0.03	0.44 ± 0.001	6.01 ± 0.01
**SC-ME2**	159 ± 0.12	22.5 ± 0.58	0.39 ± 0.002	5.90 ± 0.03
**SC-ME3**	262 ± 0.26	12.15 ± 0.42	0.45 ± 0.001	5.71 ± 0.11
**SC-ME4**	239 ± 0.62	14.35 ± 0.15	0.36 ± 0.001	5.85 ± 0.06
**SC-ME5**	138 ± 0.71	22.85 ± 0.31	0.46 ± 0.002	5.72 ± 0.02
**SC-ME6**	193 ± 0.39	32.65 ± 0.41	0.37 ± 0.002	5.93 ± 0.03
**SC-ME7**	127 ± 0.76	17.85 ± 0.12	0.41 ± 0.001	6.00 ± 0.05
**SC-ME8**	121 ± 0.25	21.85 ± 0.3	0.38 ± 0.002	5.71 ± 0.02

^a^ Values are expressed as mean ± SD.

**Table 4.** Sildenafil Citrate-Microemulsions Drug Content %, pH of Microemulsion, and Mean Droplet Size After 6 Months (N = 3)

ME Code	Drug Content (%)	pH of Microemulsion ^a^	Mean Droplet Size (nm) After 6 Months ^a^
**SC-ME1**	98.10	6.12 ± 0.01	6.24 ± 0.11
**SC-ME2**	98.02	6.01 ± 0.01	21.5 ± 0.58
**SC-ME3**	98.42	5.85 ± 0.02	12.15 ± 0. 7
**SC-ME4**	99.03	5.95 ± 0.05	15.35 ± 0.5
**SC-ME5**	98.31	5.83 ± 0.02	20.85 ± 0.51
**SC-ME6**	98.43	6.08 ± 0.01	32.65 ± 0.41
**SC-ME7**	99.21	6.1 ± 0.05	17.85 ± 0.23
**SC-ME8**	98.05	5.87 ± 0.02	21.45 ± 0. 3

^a^ Values are expressed as mean ± SD.

**Table 5.** Release Percent and Kinetic Model Release of the Cosurfactant-Microemulsions (N = 3)

MEs Code	SC Release (R_2 hours_) ^a^	SC Release (R_24 hours_) ^a^	Kinetic Model	R^2^
**SC-ME1**	1.02 ± 0.051	26.20 ± 0.020	Higuchi	0.8404
**SC-ME2**	2.69 ± 0.022	26.18 ± 0.016	Higuchi	0.8432
**SC-ME3**	2.13 ± 0.013	40.49 ± 0.035	First	0.8990
**SC-ME4**	4.81 ± 0.040	46.77 ± 0.052	Higuchi	0.8366
**SC-ME5**	1.36 ± 0.039	59.58 ± 0.018	First	0.8380
**SC-ME6**	8.32 ± 0.028	58.35 ± 0.046	Higuchi	0.8806
**SC-ME7**	3.84 ± 0.017	27.73 ± 0.022	Higuchi	0.8947
**SC-ME8**	4.54 ± 0.033	29.41 ± 0.035	Higuchi	0.8688

^a^ Values are expressed as mean ± SD.

**Table 6.** Cosurfactant-Microemulsions EX-Vivo Permeability Parameters Excised Rat Skin Model (N = 3) ^a^

ME Code	J_ss_ (mg/cm^2^.h)	D_app_ (cm^2^/h)	P (cm/h)	T_lag_ (h)	ER_flux_	ER_D_	ER_p_
**Control**	0.0002 ± 0.0001	0.006 ± 0.0001	0.02 ± 0.0001	9.51 ± 0.05	-	-	-
**SC-ME1**	0.0073 ± 0.0004	0.0993 ± 0.102	0.00073 ± 0.0004	1.169 ± 1.209	36.75 ± 0.68	16.51 ± 0.41	36.51 ± 0.69
**SC-ME2**	0.0026 ± 0.0002	0.0091 ± 0.0003	0.0002 ± 0.0002	5.895 ± 0.205	13.25 ± 0.5	1.52 ± 0.04	13.24 ± 0.05
**SC-ME3**	0.0056 ± 0.0030	0.0139 ± 0.0022	0.0005 ± 0.0003	3.97 ± 0.644	28.25 ± 7.3	2.31 ± 0.58	28.15 ± 0.08
**SC-ME4**	0.0059 ± 0.0007	0.0232 ± 0.0034	0.0005 ± 0.0007	2.360 ± 0.354	29.5 ± 0.04	3.8 ± 0. 61	29.5 ± 0.08
**SC-ME5**	0.0095 ± 0.0027	0.0500 ± 0.0078	0.0009 ± 0.0002	1.095 ± 0.172	47.75 ± 0.5	8.31 ± 0.57	47.74 ± 0.07
**SC-ME6**	0.0054 ± 0.0005	0.1457 ± 0.1768	0.0005 ± 0.0005	1.410 ± 1.711	27.01 ± 0.2	24.21 ± 0.04	27.02 ± 0.03
**SC-ME7**	0.0235 ± 0.0007	0.0255 ± 0.0072	0.0023 ± 0.0001	2.206 ± 0.623	115.25 ± 0.1	4.24 ± 0.22	115.23 ± 0.2
**SC-ME8**	0.0226 ± 0.0019	0.111 ± 0.0887	0.0022 ± 0.0001	0.704 ± 0.558	113.25 ± 0.16	18.61 ± 0.2	113.22 ± 0.33

^a^ Values are expressed as mean ± SD.

## 5. Discussion

The evaluation of SC solubility in different components of MEs indicated that specific compositions, including OA and TP as the oil phase, Tween 80 and Span 20 as the S mixture, and PG as the CS, were the optimal choices for preparing SC-MEs in this study. The phase diagrams demonstrated an expanded ME region with increasing S/CS ratios (26). Additionally, there was a significant correlation (P < 0.05) between the viscosities of MEs and the percentages of both water and oil phases, with viscosity increasing as the water content decreased and the oil phase content increased. These results align with those of a previous study by Moghimipour et al. (27). Enhancing the effective surface area through particle size reduction can improve the skin permeability and bioavailability of MEs (28). Data analysis and findings in Table 3 revealed a significant correlation between the average droplet size of MEs and the oil percentage, showing that a decrease in the oil percentage led to larger SC-ME droplets. The measurement of the PI is crucial for assessing droplet size homogeneity in each ME formulation. In all ME samples, PI values were below 0.5, indicating minimal variation in droplet size, aligning with findings from a previous study (18). According to the drug release data, reducing the oil content of SC-MEs by 5% (w/w) increased the drug release within 24 hours. Formulations such as SC-ME3, SC-ME4, SC-ME5, and SC-ME6 showed enhanced drug release, with SC-ME1 exhibiting the highest release among the formulations with a 25% (w/w) oil phase.

Moreover, the SC-ME5 formulation, containing water (10% w/w), S and CS (85% w/w), and oil (5% w/w), demonstrated the highest 24-hour release rate (R_24 hours_) at 59.58%. Notably, aside from the reduced oil content, the specific S/CS ratio and the water content significantly influence the release of active ingredients. ANOVA analysis revealed a significant correlation between the SC release rate at 2 hours (SC-R_2 hours_) and the combined S and CS variable (S + C), achieving statistical significance at a probability level of less than 0.05. Similarly, a significant relationship was observed between the oil and water percentages and the SC release rate at 24 hours (SC-R_24 hour_), indicating that a lower percentage of water and oil in SC-MEs leads to an increase in SC-R_2 hour_. The correlation coefficient between the steady-state flux (J_ss_) and the percentages of oil and water phases was statistically significant, showing that an increase in oil content and a decrease in water content resulted in a reduction in the J_ss_ parameter. Moreover, the response surface and contour plots (Figure 3A and B) showed a significant difference for the oil and water variables in their impact on the J_ss_ parameter. However, while the permeability parameter (P) exhibited a significant correlation with the percentages of oil and water phases, the apparent D_app_ did not show a significant relationship with these independent variables. Additionally, there was a significant correlation between the T_lag_ and the oil percentage, with an increase in oil content significantly raising the T_lag_ parameter. The J_ss_ and ER_flux_ parameters for the SC-ME7 formulation were 0.0235 mg/cm^2^.h and 115.25 ± 0.1, respectively, representing a 117-fold increase in J_ss_ compared to the control sample. While the ME-S-7 formulation appears to be more effective in the transdermal delivery of SC, further investigation is needed. The inclusion of an optimal amount of CS, acting as a penetration enhancer, may contribute to the higher permeability of SC through the skin, as observed with SC-ME7 (29).

Therefore, the reduced steady-state flux (J_ss_) parameter in other formulations might result from a non-optimal amount of PG despite similar oil and water proportions. Consequently, the quantity of the CS is critical in enhancing skin permeability in ME-based formulations. Similarly, in a related study, researchers asserted that the S/C mixture's content in an ME plays a pivotal role in hydroquinone's permeability through skin layers (30). Additionally, skin permeability can be influenced by various factors; for instance, PG can boost skin permeation by enhancing the extraction of lipids and proteins, increasing swelling in the outer layer of the stratum corneum, facilitating the drug's partition into the skin, and improving drug solubility (31). It has also been documented that S like Tween 80 promote the permeation of therapeutic agents across biological membranes (32). Prior studies have highlighted the effectiveness of ME carriers in enhancing dermal drug delivery (30, 33). In this study, both the J_ss_ and the permeability coefficient through rat skin increased for all SC-ME samples. Typical enhancers can enhance the skin permeation rate of drugs by optimizing permeability parameters based on specific structural properties (29). The ME systems have shown great potential for the topical administration of therapeutic agents. The benefits of MEs may stem from their excellent solubilization capacity for low-water or anhydrous solution materials and a high capacity for drug integration. Moreover, the ME system can alter the drug's partitioning into the stratum corneum layer of the skin by including drugs in the system's internal phase (34-36). Thus, ME formulations can potentially act as effective permeation enhancers due to their inherent properties, significantly improving transdermal drug delivery. Therefore, SC-ME may serve as a skin permeation enhancer to enhance SC delivery through the skin.

### 5.1. Conclusions

This study has demonstrated that the proportions of various ME components, such as oil, water, S, and CS, significantly affect its physicochemical characteristics and permeability parameters. The Higuchi and first-order models accurately describe the SC drug release from all selected SC-ME formulations, showing extended release compared to the free drug solution. The stability of SC was enhanced in ME formulations. The prepared MEs exhibited a significant increase in permeability parameters and permeation rate through rat skin.
